# Deep Recursive Bayesian Tracking for Fully Automatic Centerline Extraction of Coronary Arteries in CT Images

**DOI:** 10.3390/s21186087

**Published:** 2021-09-10

**Authors:** Byunghwan Jeon

**Affiliations:** School of Computer Science, Kyungil University, Gyeongsan 38428, Korea; bhjeon@kiu.kr

**Keywords:** coronary artery, deep learning, tracking, computed tomography

## Abstract

Extraction of coronary arteries in coronary computed tomography (CT) angiography is a prerequisite for the quantification of coronary lesions. In this study, we propose a tracking method combining a deep convolutional neural network (DNN) and particle filtering method to identify the trajectories from the coronary ostium to each distal end from 3D CT images. The particle filter, as a non-linear approximator, is an appropriate tracking framework for such thin and elongated structures; however, the robust ‘vesselness’ measurement is essential for extracting coronary centerlines. Importantly, we employed the DNN to robustly measure the vesselness using patch images, and we integrated softmax values to the likelihood function in our particle filtering framework. Tangent patches represent cross-sections of coronary arteries of circular shapes. Thus, 2D tangent patches are assumed to include enough features of coronary arteries, and the use of 2D patches significantly reduces computational complexity. Because coronary vasculature has multiple bifurcations, we also modeled a method to detect branching sites by clustering the particle locations. The proposed method is compared with three commercial workstations and two conventional methods from the academic literature.

## 1. Introduction

Extraction of coronary arteries in coronary computed tomography angiography (CCTA) is a prerequisite task for the automatic quantification of coronary lesions. In clinical application, quantification of coronary artery lesions is critical for correct diagnosis, treatment, and procedure planning. However, the quantification of coronary artery lesions still requires manual annotation by an experienced expert, which becomes a considerable burden both in time and cost. Coronary arteries are represented as a tree structure in a three-dimensional (D) volume image and elongated with an inhomogeneous contrast enhancement on the lesion. Automatic segmentation of coronary arteries in CT images remains a challenge because coronary arteries are elongated and have complex tree shapes.

In the literature [1,2], considerable attention was paid to the analysis of curvilinear or vascular structures. Seven geometric and four photometric characteristics were introduced for the definition of curvilinear objects as a region of pixels or voxels within one image [2]. Coronary arteries as curvilinear objects have the same characteristics, and the representative characteristics are:

- The region of the coronary artery is thin across a long path.
- The voxels have significantly different intensities compared to the neighboring background.
- The cross-sectional profile—the intensity values transverse to the main direction—follow a specific distribution.
- The variation in color along the main direction is smooth.
- Coronary arteries have local curvatures; for instance, some parts may be mostly straight, other parts can admit soft bends, and other parts may be highly tortuous.
- Coronary arteries have bifurcation sites that are defined as three-branch joints.

There are several approaches to locating coronary arteries based on the representative characteristics of the curvilinear objects. Lorenz et al. [3] proposed supervised wave propagation based on the models of hyper-intensity, locally tubular geometry, centerline smoothness with the adaptive threshold, and co-variance analysis of extracted segments. Lorigo et al. [4] proposed a curve evolution approach based on the centerline smoothness using curvature regularization and image gradients. Hessian-based minimal paths were found using the multiscale Hessian-based vessel enhancement filters [5,6,7].

Particle filtering-based tracking methods for coronary artery extraction were introduced by varying measurement models or using novel prior information [8,9,10,11,12,13]. Lesage et al. proposed the representative method [9,10] based on the particle filtering framework. Flux-based vascular features were utilized for the likelihood function, and medial-based geometric models were learned by kernel density estimation in terms of the scale and direction for the prior functions. This method has significant potential for improvement by employing a deep neural network as a robust observation and measurement model. In such a stochastic framework, the most important component is an accurate measurement of the vesselness, which mostly affects tracking results.

Mathematical modeling features for a coronary artery is a challenging task because coronary arteries are thin, elongated, and have complex tree structures. The image qualities can vary by the noise, artifacts, contrast injection timing, which makes the problem more challenging; however, a convolutional neural network (CNN) can extract representative features effectively [14]. CNNs have been successfully applied to vascular segmentation, and motion estimation on various image modalities including cardiac CT images [15,16,17,18,19].

Wolterlink et al. [16] proposed a CNN-based method for coronary artery tracking. This method utilizes 3D volume patches to observe the contextual information around the tracker, which can robustly predict 3D orientations; this, eventually, leads to the location of centerlines of coronary arteries. Similarly, Salahuddin et al. [19] proposed a 3D patch-based CNN method to track the coronary artery centerlines. Jung et al. [17] proposed a CNN-based method for coronary motion estimation where the 2D cross-sectional patches of coronary arteries were sampled and learned for motion correction; finally, the coronary arteries were reconstructed in a 3D volume using the compensated 2D patches. The method shows that CNN can learn the features of coronary arteries using cross-sectional patches. Zhang et al. [18] proposed a CNN-based deep reinforcement learning method. The method employed double deep Q-learning and designed Markov decision process for branch-aware centerline extraction, and shows a higher performance in processing time. Kong et al. [20] proposed a novel tree-structured convolutional gated recurrent unit model to learn the structure of coronary arteries. The method demonstrates that long short-term memory can learn the tree structures of coronary arteries. Inspired by U-Net, Chen et al. [21] proposed an architecture having multiple auxiliary branches; here, an uncertainty map is generated from the multiple abstract feature maps in the inference stage, and the uncertainty map is used to refine the segmentation results.

In this study, we propose a CNN-based stochastic tracking algorithm for the extraction of coronary arteries from 3 to D CCTA, which is inspired by recently introduced methods utilizing a spherical local image patch-based CNN [16] and the adaptive particle filtering method [10]. The proposed method uses tangent patches on a spherical surface as an input of the CNN model, and the robust vesselness probability squashed by a softmax function is utilized as a likelihood function in our particle filtering framework. Our transition priors consider variations in both direction changes and intensity distributions of patches. Further, we present the robust bifurcation detection model based on clustering the particles.

Our contribution is the development of a fully automatic method to track coronary arteries. The deep neural network is integrated with a particle filtering framework. The particles are re-used to identify the bifurcation site by clustering the particles for every time-step. In terms of accuracy, the method shows a higher performance compared with existing methods. The proposed method can eventually provide all the centerlines as a tree structure given CT images.

## 2. Methods

### 2.1. Tracking Scheme

**Figure 1:**
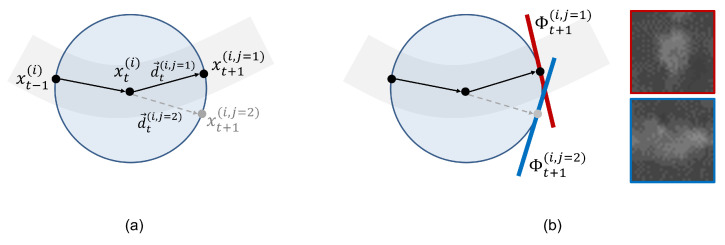
Vascular geometric model: (**a**) the edge of the sphere (drawn as circle in this figure) indicates the potential successive samples of $x_{t}^{\left(i\right)}$, e.g., $x_{t+1}^{\left(i,j\right)}$, and direction ${\vec{d}}_{t+1}^{\left(i,j\right)}$ are retrospectively fixed considering $x_{t+1}^{\left(i,j\right)}$. (**b**) The likelihood function is approximated by employing a 2D tangent patch-based convolutional neural network. The tangent patches Φ can be achieved using the direction ${\vec{d}}_{t}^{\left(i,j\right)}$ as normal and the centroid location $x_{t+1}^{\left(i,j\right)}$. When the tangent patch is well aligned to the main direction, the cross-sectional shape of the coronary artery in the patch image will be near-circular (red). Otherwise, the cross-sectional shape will be irregular (blue). A simple CNN can learn for these small patch images, and the robust vesselness measure is possible to accomplish by the trained network.

Coronary arteries found in 3D CCTA are thin, elongated, and tree-structured objects. Our system aims at recovering the vascular centerlines with the tree structure as a chain of sphere centers $X_{T}=\left\{x_{0},\ldots ,x_{T}\right\}$ that are estimated given the observation and the stationary image $Y_{t}=Y,\forall t$. The recursive fashion of Maximum a Posteriori (MAP) estimation is a feasible solver for the most probable path of the coronary artery [22]. Thereafter, a Monte-Carlo approximation is varied out for the posterior distribution $p(X_{t}|Y)$ using the weighted population of $N_{t}$ discrete samples $S_{t}={\left\{x_{t}^{\left(i\right)},w_{t}^{\left(i\right)}\right\}}_{i=1}^{N_{T}}$. A weight $w_{t+1}^{\left(i,j\right)}$ is evaluated from the posterior distribution in Equation (1). The state $x_{t}^{\left(i\right)}$ is a discrete location at time-step *t*, and $x_{t+1}^{\left(i,j\right)}$ denote the potential successors of $x_{t}^{\left(i\right)}$. When considering $x_{t+1}^{\left(i,j\right)}$, the orientation ${\vec{d}}_{t}^{\left(i,j\right)}$ can be retrospectively fixed as shown in Figure 1a, and the associated weight $w_{t+1}^{\left(i,j\right)}$ is computed as follows:

(1) $$w_{t+1}^{\left(i,j\right)}\propto w_{t}^{\left(i\right)}p\left(Y|x_{t+1}^{\left(i,j\right)}\right)p\left(x_{t+1}^{\left(i,j\right)}|x_{t}^{\left(i\right)}\right)$$

where $p(Y|x_{t+1}^{\left(i,j\right)})$ and $p(x_{t+1}^{\left(i,j\right)}|x_{t}^{\left(i\right)})$ are the likelihood and the transition prior, respectively. Using the principle of sequential importance resampling (SIR) at every time-step *t*, we estimated the posterior distribution in two steps: prediction and update. In the prediction step, the $N_{T}$ samples $\left\{x_{t+1}^{\left(i,j\right)},w_{t+1}^{\left(i,j\right)}\right\}$ are drawn from the previous distribution $\left\{x_{t}^{\left(i\right)},w_{t}^{\left(i\right)}\right\}$. In the update step, the samples are weighted by Equation (1). The result would be the weighted new population $\left\{x_{t+1}^{\left(i\right)},w_{t+1}^{\left(i\right)}\right\}$. For the centerline estimation, we can estimate and update the state $x_{t}$ as,

(2) $${\hat{x}}_{t}={\hat{x}}_{t-1}+\gamma \sum_{i=1}^{N_{T}}w_{t-1}^{\left(i\right)}{\vec{d}}_{t-1}^{\left(i\right)}$$

where γ denotes the step size, and $\left|\right|{\vec{d}}_{t-1}^{\left(i\right)}\left|\right|=1$.

### 2.2. Likelihood Approximator $p(Y|x_{t+1}^{\left(i,j\right)})$

**Figure 2:**
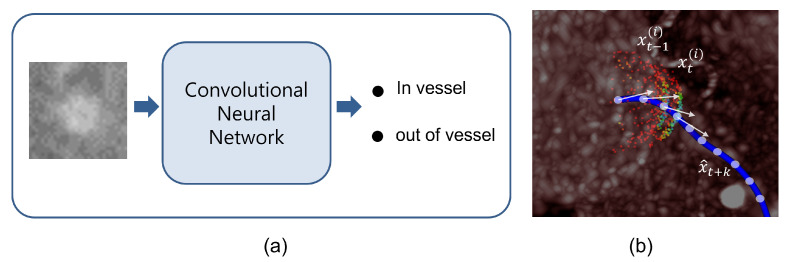
(**a**) The local tangent patch images are used for the input of CNN used for classification into ‘in-vessel’ and ‘out-of-vessel’, and (**b**) the samples on spheres with two steps and their measured weights are also visualized with a color scale (blue-red). The ground truth is co-visualized (blue line).

Our likelihood function is designed to provide reliable and robust vesselness probabilities. The likelihood function is approximated by the local patch-based CNN in Figure 2a. We utilize the softmax values of the CNN to obtain the vesselness probabilities. Considering a successoral location $x_{t+1}^{\left(i,j\right)}$, the tangent patch images $\Phi_{t}^{\left(i,j\right)}$ can be achieved, where the patch’s centroid and the normal are $x_{t+1}^{\left(i,j\right)}$ and ${\vec{d}}_{t}^{\left(i,j\right)}$. Then,

(3) $$p\left(Y|x_{t+1}^{\left(i,j\right)}\right)\approx p\left(y=1|f_{\theta }\left(\Phi_{t}^{\left(i,j\right)}\right)\right)=\mathrm{Softmax}{\left(f_{\theta }\left(\Phi_{t}^{\left(i,j\right)}\right)\right)}_{\mathrm{IN}-\mathrm{VESSEL}}$$

where $f_{\theta }$ is a deep CNN parametrized by θ. The parameter θ is highly optimized by training with 170,000 local patch images as described in detail in Section 3.1. As shown in Figure 1b, the cross-sectional shape of the coronary artery is near-circular when the normal of the tangent patch is well aligned to the main direction of the coronary artery; otherwise, the cross-sectional shapes of coronary arteries appear irregular. The original purpose of the CNN for the training was to classify the patches into in-vessel (y=1) and out-of-vessel (y=0) classes. We directly used the output of the softmax function in Equation (3) for the likelihood term and in Equation (1) for our tracking scheme.

### 2.3. Transition Prior $p(x_{t+1}^{\left(i,j\right)}|x_{t}^{\left(i\right)})$

Our transition prior is assumed to be the first-order Markovian model in Equation (4). We mainly have two priors with independent variables considering direction variations and the similarity of the adjacent patch-images for vascular dynamics.

(4) $$\begin{array}{cc} p(x_{t+1}^{\left(i,j\right)}|x_{t}^{\left(i\right)}) & =p({\vec{d}}_{t}^{\left(i,j\right)},\Phi_{t}^{\left(i,j\right)}|{\vec{d}}_{t-1}^{\left(i\right)},\Phi_{t-1}^{\left(i\right)})\\ \; & =p\left({\vec{d}}_{t}^{\left(i,j\right)}|{\vec{d}}_{t-1}^{\left(i\right)}\right)p\left(\Phi_{t}^{\left(i,j\right)}|\Phi_{t-1}^{\left(i\right)}\right) \end{array}$$

where $p({\vec{d}}_{t}^{\left(i,j\right)}|{\vec{d}}_{t-1}^{\left(i\right)})$ and $p(\Phi_{t}^{\left(i,j\right)}|\Phi_{t-1}^{\left(i\right)})$ in Equation (4) are the prior functions for directional changes and the intensity variation of the patch images between adjacent time-steps.

**Figure 3:**
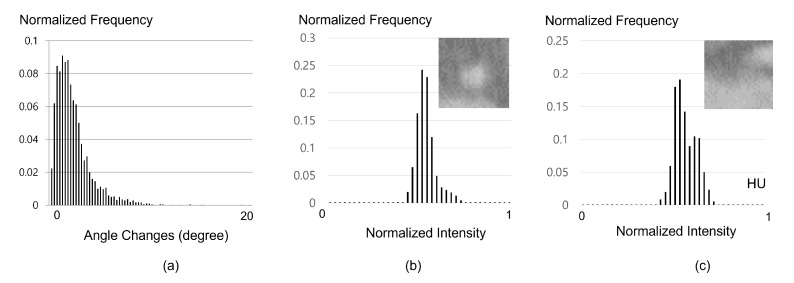
As prior information, the tracker prefers to change the directions smoothly so that the image distribution between adjacent time-steps do not change significantly. A directional prior distribution learned from ground truth (**a**) can be directly used in our method, and the local patches are simply converted to an intensity distribution form (**b**,**c**) for distance measure using Jensen–Shannon divergence.

From the GT centerlines in our database, the angle values by $<{\vec{d}}_{t},{\vec{d}}_{t+1}>$ can be sampled to construct a detailed histogram; furthermore, the $p({\vec{d}}_{t}^{\left(i,j\right)}|{\vec{d}}_{t-1}^{\left(i\right)})$ term is directly valuated using the learned angle histogram as shown in Figure 3a.

Furthermore, the patch images have an intuitive feature in that the coronary arteries appear circular, whereas the local intensity distributions from the proximal to distal parts of coronary arteries do not change significantly. Based on these characteristics, we employed Jensen–Shannon divergence to measure the distance between two local image distributions as,

(5) $$\begin{array}{c} D\left(P||Q\right)=\frac{1}{2}KL\left(P||M\right)+\frac{1}{2}KL\left(Q||M\right) \end{array}$$

where $KL\left(P||Q\right)=\sum_{x\in X}P\left(x\right)log\left(\frac{P(x)}{Q(x)}\right)$ and $M=\frac{1}{2}\left(P+Q\right)$. Distributions *P* and *Q* in Equation (5) are the normalized image histograms $\Pi_{t}^{\left(i,j\right)}$ and $\Pi_{t-1}^{\left(i\right)}$ converted from the local patches $\Phi_{t}^{\left(i,j\right)}$ and $\Phi_{t-1}^{\left(i\right)}$ as shown in Figure 3b,c. D(P||Q) can have [0,1] values for a similarity distance, with values near zero indicating a similarity between distributions and positive values indicating divergence in distribution. Thereafter, we mapped the distance values to a weighting function [23]:

(6) $$\begin{array}{c} w_{\Phi }\left(x\right)=\left\{\begin{array}{cc} 2{\left(x\right)}^{3}-3{\left(x\right)}^{2}+1, & \mathrm{if}\;0\leq x\leq 1\\ 0, & \mathrm{otherwise} \end{array}\right. \end{array}$$

$w_{\Phi }$ in Equation (6) is a polynomial function to approximate the probability given the distribution similarity as,

(7) $$\begin{array}{c} p\left(\Phi_{t}^{\left(i,j\right)}|\Phi_{t-1}^{\left(i\right)}\right)=w_{\Phi }(\lambda D(\Pi_{t}^{\left(i,j\right)}\left|\right|\Pi_{t-1}^{\left(i\right)})) \end{array}$$

where λ(=6) is a scale factor for the probability calibration. Overall, our transition prior $p(x_{t+1}^{\left(i,j\right)}|x_{t}^{\left(i\right)})$ in Equation (7) prefers that the direction and intensity distribution do not change significantly.

### 2.4. Majority-Minority (M&m) System for Bifurcation Detection

**Figure 4:**
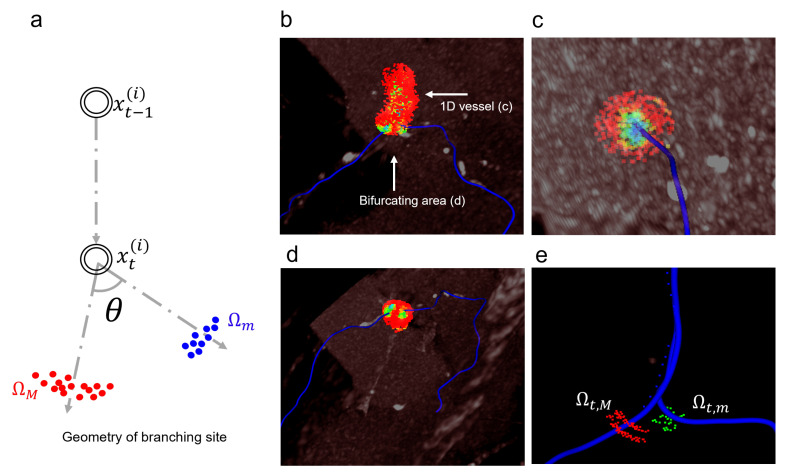
(**a**) A geometry of bifurcating site. (**b**) Particles and the posterior distribution are visualized after multiple tracking steps. (**c**) Posterior distribution during tracking in 1-D vessel structure. (**d**) Posterior distribution on branching site. (**e**) Important split particles.

In the case of vessels with a single structure, the posterior distribution is clearly higher around the center of the vessel; an example can be seen in Figure 4c. However, at the branching point, higher values are mapped in two places, as seen in Figure 4d. Thus, it is not easy to determine the direction of the main vessel at the branching site. For every step *t*, the proposed method utilizes only the important samples $\Omega_{t}\subset S_{t}$, leading to a geometrical splitting of the important samples into K=2 clusters $\Omega_{t,k}\subset \Omega_{t}$, $\Omega_{t,k}={\left\{w_{t,k}^{\left(i\right)},c_{t,j}^{\left(i\right)}\right\}}_{i=1}^{\left|\right|\Omega_{t,j}\left|\right|}$ by density-based spatial clustering of applications with noise (DBSCAN) [24] visualized in Figure 4e. The weighted average $\mu_{t,k}=\Sigma_{i=1}^{\left|\right|\Omega_{t,k}\left|\right|}w_{t,k}^{\left(i\right)}c_{t,k}^{\left(i\right)}$ becomes the centroid of cluster $\Omega_{t,k}$, where the weight $w_{t,k}^{\left(i\right)}$ is normalized such that $\Sigma_{i=1}^{\left|\right|\Omega_{t,k}\left|\right|}w_{t,k}^{\left(i\right)}=1$. Let ${\vec{v}}_{t,k}=\mu_{t,k}-{\hat{x}}_{t}$ be the candidate direction. The *K* directions ${\vec{v}}_{t,k}$ are used to determine whether the state is on a branching site or not by computing the angle $\theta =cos^{-1}\langle {\vec{v}}_{t,k=1},{\vec{v}}_{t,k=2}\rangle$. If the θ is higher than a specific angle α, then the current state is on a branching site.

**Figure 5:**
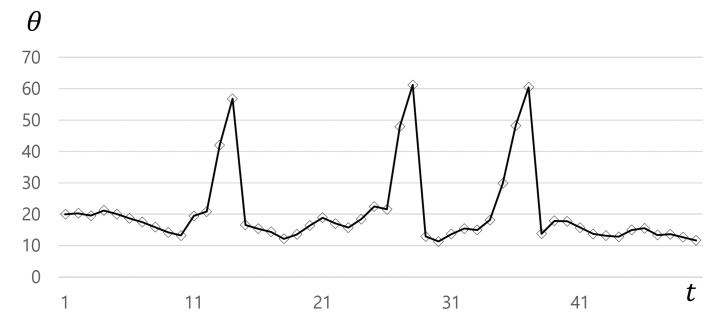
Angle trends along the centerline: the peaks represent branching sites.

The θ responses along the trajectory during tracking are plotted in Figure 5. There are some high peaks when the tracker passes branching sites, compared to a weak signal and small variations in other places. At such branching sites, the tracker continues in the main vessel direction depending on the size of clusters. The tracker recognizes the direction of the main vessel using the bigger cluster $\Omega_{t,M}$ while storing the direction using the smaller cluster $\Omega_{t,m}$ in the stack for the branch vessels, as shown in Figure 4a. $\Omega_{t,m}$ are used for the new seed points for branch vessels. The process for tracking multiple vessels as a tree structure is fully automatically performed.

### 2.5. Stopping Criterion

**Figure 6:**
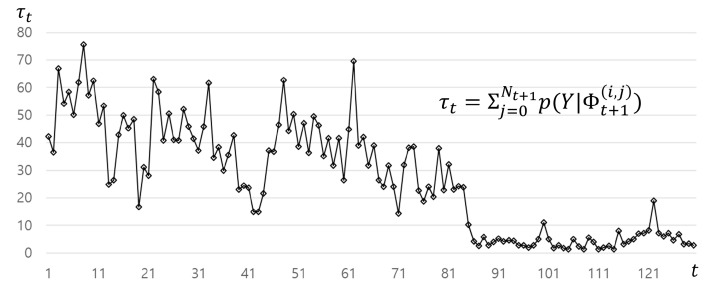
The responses of $\tau_{t}$ along the centerline: the low values with small variations are observed after around t=85.

We introduced a likelihood measurement $p(Y|\Phi_{t}^{\left(i\right)})$ in Equation (3), which purely indicates a probability of vesselness. We assume that the summation of the likelihoods measured from all particles at the coronary distals converges to zero, as the tracking goes to more distal parts. We used this assumption as a stopping criterion $\tau_{t}=\sum_{i=0}^{N_{t}}p\left(Y|\Phi_{t}^{\left(i\right)}\right)$ where $0\leq \tau_{t}\leq N_{t}$. The algorithm is forced to track the coronary artery from its ostium to time step (t=130) to check how $\tau_{t}$ changes on every time-step *t*. As shown in Figure 6, clearly different trends are observed from where a coronary exists to where a coronary does not exist. We terminate the tracking when $\tau_{t}<\beta$.

## 3. Experiment and Result

The particle filtering framework with CNN (Deep-PF) was implemented in Python using TensorFlow library. Experiments were performed using an NVIDIA Titan Xp GPU. Our framework extracts a single vessel at an average of 7.6 s. Our method was evaluated and compared with the three commercial workstations (Xelis Cardiac 3D, by INFINITT [25], and Vitrea, by vital image [26], QAngioCT, by Medis [27]) and the recently introduced stochastic methods (AS [28], AAPF [10]) on eight CT images with thirty two coronary vessels provided in MICCAI 2008 Coronary Artery Tracking Challenge (CAT08) dataset [29]. Note that it was difficult to compare AAPF directly, as it was tested on 50 private cases. For each dataset that was tested, the network parameter and prior distribution were learned on the remaining seven cases using Leave-One-Out rule.

### 3.1. Training Patch-Based CNN

To train the patch-based CNN introduced in Section 2.2, we collected 170,000 local patches from the centerline ground truth (GT) [29]. From each image, four types of vessels (RCA, LCA, LCX, and one branch) were annotated by two medical experts in GT. A single vessel consisted of centerline locations and radiuses for time-step *t* as ${\left\{x_{t},y_{t},z_{t},r_{t}\right\}}_{t=0:L}$.

**Figure 7:**
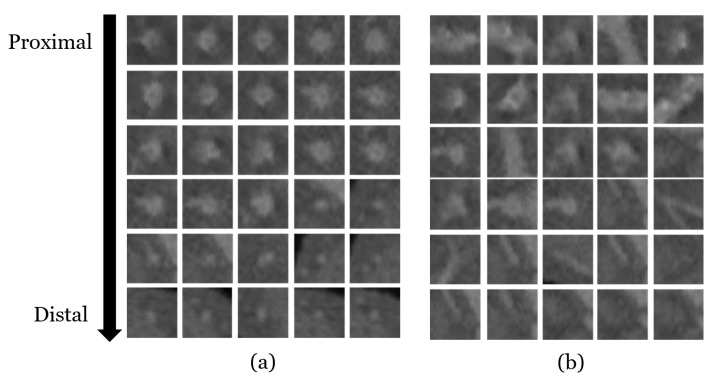
Examples of tangent planes: (**a**) The cross-sections of coronary arteries are near circular in the patches that are well aligned to the main direction of coronary arteries. (**b**) The cross-sections of coronary arteries have arbitrary shapes in the patches that are not aligned to the main direction of coronary arteries.

An even number of local patches from the proximal (t≈0) to distal parts (t≈L) are sampled for all vessel scales as shown in Figure 7a. At a specific time-step *t*, the local patch images are randomly sampled considering the system noise ϕ∼U[0,2π), and $\theta ∼N(0,\sigma_{\theta }^{2})$ for the sphere parameters. We labeled y=1 for the patches whose centroids lie inside the vessels of radius $r_{t}$; otherwise, we labeled them as y=0. Then, 85,000 patch images were evenly sampled for each class for the class balance. In total, 170,000 local patch images were sampled for training data by varying vessel types, radius, and patients. For example, LAD, LCX, RCA, a branch can be one of the vessel types, and the radius scale can vary from 2.5 mm to 0.8 mm.

A simple CNN architecture was employed for learning the local patch images, and the architecture required a 32 × 32 of input image, three convolution layers with 32, 64 and 128 channels, and three fully connected layers with 512, 256 and 2 neurons for binary classification. ReLU activation function was used for all the layers.

We trained this simple CNN with 170,000 local patches, and used the softmax values as uncertainties for the likelihood function in Equation (3). Training for the CNN model takes about 90 minutes with the GPU, TITAN XP 12GB.

### 3.2. Initialization and Parameters

The method is automatically initialized using the seed points (coronary ostia) as $x_{0}$ and $x_{1}$, and an initial main direction as ${\vec{d}}_{0}=x_{1}-x_{0}$ by the identification method [30]. We fixed the other parameters as $N_{T}=200$, λ=6, $\alpha =50^{\circ }$, β=5 for the experiment.

### 3.3. Evaluation on a CCTA Database

The robustness and accuracy were evaluated based on the criteria and the public dataset introduced in [29]. The proposed method was applied on eight patients, 32 vessels for the quantitative evaluation, and the dataset is described in Table 1. From each image, GT centerlines of four types of vessels (RCA, LCA, LCX, and one branch) are included. Each image is reconstructed to 512 × 512 × [297, 423] voxels with the range of isotropic voxel size from (0.287 mm × 0.287 mm × 0.287 mm) to (0.371 mm × 0.371 mm × 0.371 mm). The CCTA images in this public dataset are focused on hearts, and the distributions of the Hounsfield unit (HU) for regions of coronary arteries are similar for all images thanks to the contrast injection.

For the evaluation metrics [29], a point of the GT centerline is marked as true positive reference (TPR) if the distance to at least one of the points connected on the centerline result by a method to be evaluated is less than the radius, otherwise, a point of the centerline GT is marked as false negative (FN). Then, a point of the centerline result by a method is marked as true positive method (TPM) if there is at least one point on the GT centerline at a distance less than the radius defined at the GT point; otherwise, the point of the centerline result is marked as false positive (FP). Then, the evaluation metrics are defined as below,

- OV: Total overlap, $\frac{\left||TPM||+||TPR|\right|}{\left||TPM||+||TPR||+||FN||+||FP|\right|}$.
- OT: OV of the extracted centerline with the clinically relevant part of the vessel (radius ≥ 1.5 mm), which indicates how well the method is able to track the section of the vessel that is assumed to be clinically relevant. Vessel segments with a diameter of 1.5 mm or larger are assumed to be clinically relevant [31,32].
- AI: The average inside accuracy metric (AI) measures the average distance between the reference, $A\left(x\right)=\sqrt{1/n\Sigma {\left(d\left(p\left(x\right),p_{i}\right)\right)}^{2}}$, and extracted centerline for automatically extracted points that are within the radius of the reference centerline.

## 4. Evaluation and Results

**Figure 8:**
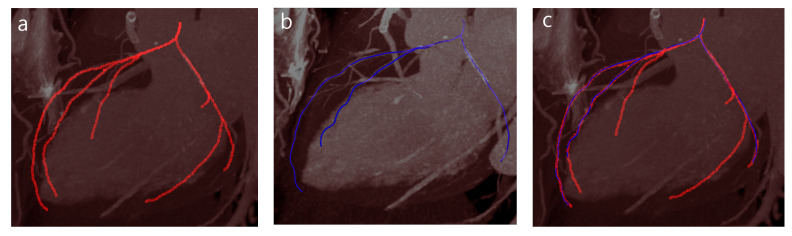
(**a**) The results of tree extraction by the proposed method have not only main coronary artery but also some small branches, (**b**) ground truth has only three main coronary arteries, and (**c**) ground truth and the result by Deep-PF is visualized.

**Figure 9:**
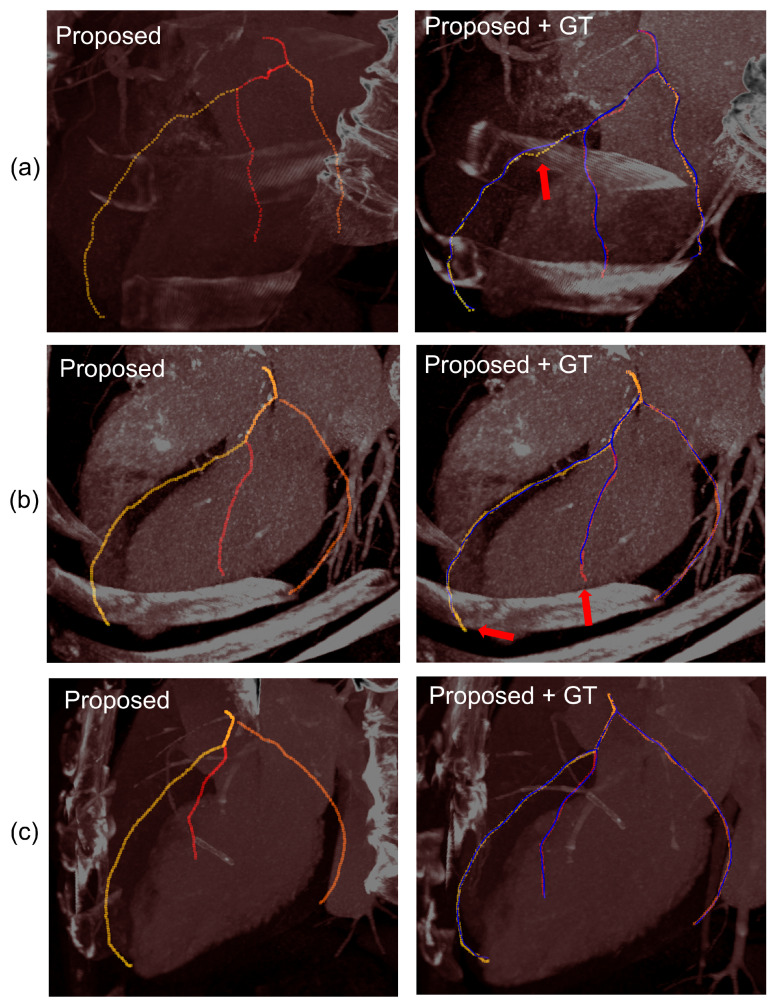
The results of three example cases (top) and overlap with ground truth (bottom, blue): (**a**) some FP area in the middle of the detected trajectory (**b**) the excessively tracked coronary artery (**c**) the well detected case.

In this study, we proposed a deep CNN with particle filtering method (Deep-PF) for extraction of coronary arteries from CT images. Table 1 shows the average overlap and accuracy results for each of the eight datasets. In terms of overlap, Deep-PF obtained an average OV of 92% and an average OT of 93%. In terms of accuracy, Deep-PF obtained AI of 0.36 mm, which is similar to the typical width of a voxel, but smaller than the spacing between slices in the dataset. The extracted coronary tree by Deep-PF and the GT centerlines are co-visualized in Figure 8, and some examples from the results are visualized in Figure 9.

Three workstations were compared with Deep-PF in Table 2. In the case of Vitrea [26], the centerline is not provided directly; however, it provides the segmentation region of the coronary artery without any user-interaction. The centerlines were manually obtained based on the automatically extracted segmentation regions. Therefore, the OV and OT corresponding to the length of the detected region are comparable. In the case of xelis [25], the centerlines were extracted based on the semi-automatic algorithm, and we gave multiple seed points. In the case of QAngioCT [27], the centerlines were extracted without any interaction. It was possible to make a comparison by referring to the method AS (active search) [28], which employed the experiment with the same measures and dataset. In the comparison presented in Table 2, the Deep-PF showed the best performance in OV and OT, which is a measure of the extent to which the coronary artery is tracked. In terms of accuracy, all methods have small values (0.23 mm–0.36 mm), which is smaller than the typical width of a voxel or similar to one in the dataset, and the AAPF method showed the highest accuracy (AI). However, the accuracy of Deep-PF can be improved by centerline refinement through post-processing. Overall, Deep-PF shows a higher performance of coronary artery extraction as an approach combining Deep CNN with particle filtering.

## 5. Discussion

In this paper, we have presented a method for a robust centerline extraction method using CNN and particle filtering framework. Tangent patches reflect the features of the cross-sectional shape of coronary arteries that are circular in appearance. CNN can learn these features and provide a robust vesselness probability. Our particle filtering framework is a novel design that uses CNN as a likelihood function. Furthermore, we have presented a robust bifurcation detection model by clustering the particles. The proposed method can automatically extract all the centerlines as a tree structure.

AAPF [10] is based on a highly optimized coronary artery tracking approach by improving the original particle filtering framework. However, AAPF still uses image-gradient-based hand-crafted features for the likelihood function. On the other hand, the proposed method uses CNN to measure vesselness probability with a more sophisticated and large number of features. However, since both methods use local information sequentially, it is very challenging near the distal part of vessels. The proposed method showed slightly higher performance by minimizing the problem of early stopping or over-tracking by reducing uncertainty at the distal part of the vessels.

In recent studies, Wolterink et al. [16] proposed a 3D patch-based CNN that independently classifies the local orientation of a coronary artery, and the method shows the accurate performance. On the other hand, the proposed method is designed to minimize the computation cost to evaluate each particle using 2D patches since particle filtering is a population-based approach. From the perspective of observing image information. In fact, the quantity of the contextual information from the multiple 2D tangent patches is not small compared to a single 3D boxed patch. The use of a 3D boxed patch has the advantage to observe the structural contextual information around the tracker, while the use of multiple 2D tangent planes allows the tracker to observe the contextual information around a wider area. However, there is a clear difference in the tracking approach between the 3D patch-based CNN [16] and the proposed method. In the case of 3D patch-based CNN, a local direction with the highest probability is directly chosen by a single 3D patch for each step, and the direction is not dependent on the previous direction. Whereas the proposed method estimates an optimal direction with multiple particles and the particle filtering framework enables the first-order Markovian property to consider the previous states, which can prevent leakages to other organs or non-coronary vessels.

A limitation of the proposed method may arise when the coronary arteries have long missing regions due to severe complex coronary lesions. The tracker may terminate earlier, which is critical for achieving an accuracy score. This is actually a common limitation that may occur in other tracking-based methods. Even though the proposed method shows a higher performance compared with other tracking-based methods, it has the same limitations. Furthermore, the prior functions in our method make the trajectories smoother, which leads the tracker to be generally more robust. In spite of this advantage, the tracker may leak to other organs if there are very sharply curved vessels. Furthermore, detecting the terminal point is a common challenge of all tracking-based methods because these methods eventually find the local maximal path. To improve these concerns, a graph-based global approach may be an alternative, but there is a limit to its practical use because the computation cost is too high. An eclectic alternative may be policy-based reinforcement learning methods that approximate a global solution.

Deep reinforcement learning (DRL) is one feasible solver for the sequential decision process. DRL can ideally learn and search near-global paths quickly, which is demonstrated from the literature [33,34,35,36]; however, it is still not simple to learn every scenario of the coronary arteries, thus its accuracy is still incomparable with the state-of-the-art methods [18]. The learned policy by DRL, nevertheless, may be helpful for tracking-based methods by hybridizing local and global perspectives.

## 6. Conclusions

In this study, we proposed a deep learning-based tracking method for the extraction of coronary arteries from CT images. The proposed method shows best performances as 0.92, 0.93 and 0.36 for OV, OT, AI, respectively. However, this approach still offers potential for improvement. We are currently improving our method to combine with the policy-based guidance from deep reinforcement learning, and planning to test the method with more CT images to validate its robustness.

## Figures and Tables

**Table 1:** Results of the centerline extraction from CAT08 training set.

Dataset	Image Details and Measures				
	Image Quality	Calcium Score	OV	OT	AI
0	Moderate	Moderate	0.93	0.93	0.27
1	Moderate	Moderate	0.93	0.94	0.49
2	Good	Low	0.92	0.87	0.34
3	Poor	Moderate	0.90	0.92	0.48
4	Moderate	Low	0.97	0.98	0.31
5	Poor	Moderate	0.89	0.89	0.29
6	Good	Low	0.95	0.97	0.47
7	Good	Severe	0.83	0.85	0.38
Average			0.92	0.93	0.36

**Table 2:** Comparison with other methods including commercial workstations. All the methods are compared with the same public dataset, 8 patients, 32 vessels, described in Table 1, except the AAPF method. AAPF used 51 private CT images.

		Solver					
		QAngioCT [27]	Xelis [25]	Vitrea [26]	AS [28]	AAPF [10]	Deep-PF
Measure	OV	0.86	0.78	0.86	0.84	0.86	0.92
	OT	0.88	0.80	0.89	0.88	0.92	0.93
	AI	0.36	-	-	-	0.25	0.36

## Data Availability

Not applicable.
